# Dr. William Webb Browning

**Published:** 1900-12

**Authors:** 


### OBITUARY NOTES.

Dr. William Webb Browning, of Brooklyn, died at his home October 3, 1900, of apoplexy, aged 48 years. Dr. Browning was first a lawyer and then a doctor, graduating in medicine at Bellevue in 1884. He was professor of anatomy and clinical orthopedics at Long Island College Hospital, at the time of his death, and he had been a writer of considerable renown.

